# Prohibitin 2 localizes in nucleolus to regulate ribosomal RNA transcription and facilitate cell proliferation in RD cells

**DOI:** 10.1038/s41598-018-19917-7

**Published:** 2018-01-24

**Authors:** Zilong Zhou, Huihan Ai, Kun Li, Xinlei Yao, Wenbin Zhu, Lei Liu, Chunlei Yu, Zhenbo Song, Yongli Bao, Yanxin Huang, Yin Wu, Lihua Zheng, Ying Sun, Guannan Wang, Kewei Ma, Luguo Sun, Yuxin Li

**Affiliations:** 1 National Engineering Laboratory for Druggable Gene and Protein Screening, Northeast Normal University, Changchun, 130024 China; 2 Institute of Genetics and Cytology, Northeast Normal University, Changchun, 130024 China; 3 Oncology Center, First Hospital, Jilin University, Changchun, 130021 China; 4 Present Address: Research Institute of Medical Science and Pharmacy, Qiqihar Medical University, Qiqihar, 161006 China

## Abstract

Prohibitin 2 (PHB2), as a conserved multifunctional protein, is traditionally localized in the mitochondrial inner membrane and essential for maintenance of mitochondrial function. Here, we investigated the role of PHB2 in human rhabdomyosarcoma (RMS) RD cells and found substantial localization of PHB2 in the nucleolus. We demonstrated that PHB2 knockdown inhibited RD cell proliferation through inducing cell cycle arrest and suppressing DNA synthesis. Meanwhile, down-regulation of PHB2 also induced apoptosis and promoted differentiation in fractions of RD cells. In addition, PHB2 silencing led to altered nucleolar morphology, as observed by transmission electron microscopy, and impaired nucleolar function, as evidenced by down-regulation of 45S and 18S ribosomal RNA synthesis. Consistently, upon PHB2 knockdown, occupancy of c-Myc at the ribosomal DNA (rDNA) promoter was attenuated, while more myoblast determination protein 1 (MyoD) molecules bound to the rDNA promoter. In conclusion, our findings suggest that nucleolar PHB2 is involved in maintaining nucleolar morphology and function in RD cells by regulating a variety of transcription factors, which is likely to be one of the underlying mechanisms by which PHB2 promotes tumor proliferation and represses differentiation. Our study provides new insight into the pathogenesis of RMS and novel characterizations of the highly conserved PHB2 protein.

## Introduction

Rhabdomyosarcoma (RMS) is the most prevalent soft tissue sarcoma in children and adolescents, accounting for 5% of all pediatric tumors and over 40% of pediatric soft tissue sarcomas^1^. RMS can be grouped into two major histological subtypes, embryonal (ERMS) and alveolar (ARMS) rhabdomyosarcoma^2^. ERMS represents approximately 70% of all childhood RMS, mainly affecting the head and neck and genitourinary regions^3^. Advances in the treatment of RMS have promoted the 5-year survival rate from 25% up to approximately 70%^4^. However, a high rate of metastases, drug resistance and tumor recurrence remain to be overcome. The lack of directed therapies against RMS emphasizes the need to further illuminate the molecular underpinnings of the disease.

Myogenic differentiation arrest is a main characteristic in RMS^5^. Accompanied by incomplete differentiation, cell proliferation is no longer arrested in these tumor cells^2^. The skeletal muscle differentiation process is conserved in evolution and involves an orderly cascade of events dependent on the activities of two families of transcription factors, myogenic regulatory factors (MRFs) and the myocyte enhancer factor-2 (MEF2)^6^. MRFs, which include MyoD, myogenin, myogenic factor 5 (Myf5) and MRF4, synergistically cooperate with MEF2 to induce muscle-specific gene transcription and ultimately the onset of myogenesis. Among those factors, MyoD is considered to be muscle-determining, and the expression of myogenin is a typical early myogenic differentiation marker. Although RMS cells express MyoD and myogenin, they fail to achieve complete myogenesis under differentiation conditions via mechanisms that are not fully understood. Previous studies have associated higher expression of myogenic markers with better prognosis in children with RMS^7^. Therefore, gaining a better understanding of pathogenesis that contribute to the proliferation and growth of RMS is critical.

PHB2, also designated as B-cell receptor-associated protein 37 (BAP37) and repressor of estrogen receptor activity (REA), is a highly conserved protein found in fungi, plants and humans^8,9^. Mainly, PHB2, together with PHB1, forms the eukaryotic mitochondrial prohibitin complex which is essential for mitochondrial morphogenesis and genome stability^10,11^. In addition, PHB2 has been implicated in diverse mitochondria-related functions, such as cell proliferation, cell death and aging^12^. However, its diverse functions in other cellular compartments have also been widely reported, such as in the nucleus and on the membrane^13,14^. We previously demonstrated that PHB2, as a repressor, could inhibit muscle differentiation by recruiting histone deacetylase 1 (HDAC1) to repress the transcriptional activity of both MyoD and MEF2^15^. However, whether PHB2 is involved in the development and progression of RMS has not been reported.

Our current study aimed to explore the role of PHB2 in RMS by using the RNA interference (RNAi) technique. We found that PHB2 knockdown inhibited the proliferation of the (human RMS) RD cell line, as evidenced by arrest of cell cycle and reduction of DNA synthesis. Meanwhile, PHB2 knockdown also induced apoptosis in a fraction of the cells and enhanced the differentiation of RD cells to some extent. More importantly, we observed that a substantial amount of PHB2 was localized in the nucleolus which has never been reported before. We further showed that nucleolar PHB2 might be involved in maintaining the structure and function of the nucleolus in RD cells partly by modulating the transcription of rDNA through regulating the accessibility of c-Myc and MyoD to the rDNA promoter. In summary, our results suggest a pro-tumorigenic role for PHB2 in human RMS at least partly through its specific localization in the nucleolus.

## Results

### PHB2 knockdown by siRNA inhibits RD cell proliferation

**Figure 1:**
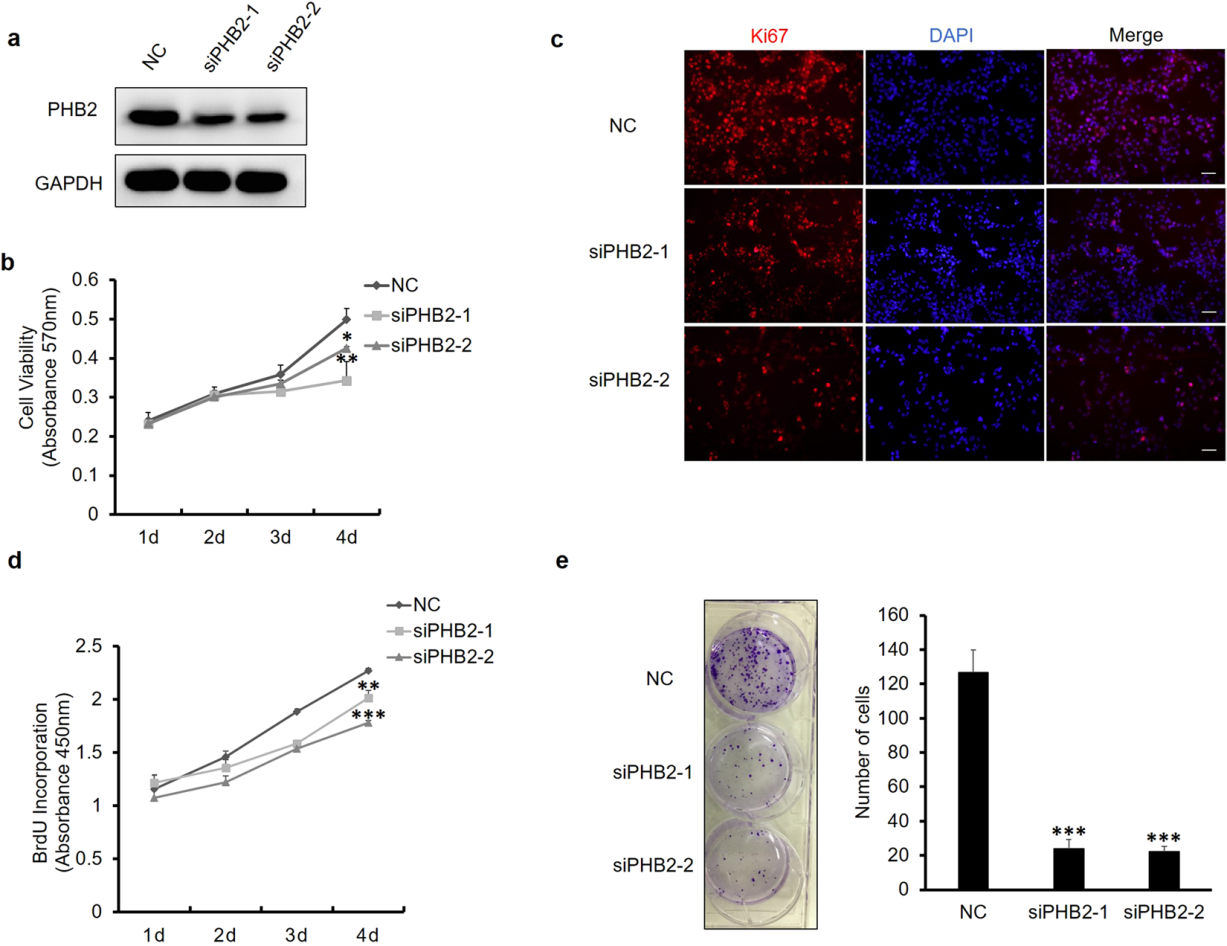
PHB2 knockdown by siRNA inhibits RD cell proliferation. (**a**) Evaluation of PHB2 knockdown by siRNA. RD cells were transfected with siRNAs against PHB2 (siPHB2-1 and siPHB2-2) or negative control (NC). Thirty-six hours later, cells were lysed and subjected to Western blotting using an anti-PHB2 antibody, and GAPDH was detected as a loading control. Full-length blots are presented in Supplementary Fig. S1. Results are representative of three independent experiments. (**b**) Effect of PHB2 knockdown on cell viability in RD cells. RD cells in 6-well plates were transfected with siRNAs. Thirty-six hours later, the cells were re-plated in triplicate in 96-well plates at 5000 cells per well. Cell viability was determined by the MTT assay. Data are presented as the mean ± S.D. (*n* = 3). Results are representative of three independent experiments. (**c**) Effect of PHB2 knockdown on expression of Ki-67. RD cells were fixed and stained 48 h after siRNA transfection and then monitored by immunofluorescence assay. Results are representative of three independent experiments. *Scale bar* 100 μm. (**d**) Effect of PHB2 knockdown on BrdU incorporation of RD cells. RD cells in 6-well plates were transfected with siRNAs. Twenty-four hours later, RD cells were re-plated in triplicate in 96-well plates at 5000 cells per well. Detection every 24 hours. Six hours before detection, RD cells were incubated with 10 µl/well of 10 µM BrdU labeling solution. Data are presented as the mean ± S.D. (*n* = 3). Results are representative of three independent experiments. (**e**) Effect of PHB2 knockdown on colony formation of RD cells. RD cells transfected with siRNA were plated in triplicate in 6-well plates at 1500 cells per well. After 10 days, images of the wells were taken. Data are presented as the mean ± S.D. (*n* = 3), *P < 0.05, **P < 0.01, ***P < 0.001, two-tailed Student’s t test. Results are representative of three independent experiments.

To explore the functional role of PHB2 in RMS, we first knocked down endogenous PHB2 in RD cells, an ERMS cell line, using siRNA. As shown in Fig. 1a, two separate siRNAs targeting PHB2 could both effectively down-regulate the expression of PHB2 of RD cells. Thereafter, we determined the effect of PHB2 siRNAs on RD cell proliferation in a time course experiment. Results of the MTT assay showed that RD cells transfected with PHB2 siRNAs displayed a reduced proliferation rate compared with cells transfected with the negative control siRNA (Fig. 1b). We further demonstrated that the expression of antigen Ki67, a nuclear protein associated with cell proliferation, also substantially decreased in RD cells transfected with PHB2 siRNAs (Fig. 1c). As DNA synthesis is directly related to the ability of cells to proliferate, we also examined DNA synthesis in siRNA-transfected RD cells by using a BrdU incorporation assay. Similarly, BrdU incorporation was decreased in RD cells treated with PHB2 siRNAs compared with control cells (Fig. 1d). Next, we investigated whether PHB2 silencing would also impact the clonogenic ability of RD cells. The results showed that PHB2 knockdown significantly reduced the colony forming efficiency of RD cells (Fig. 1e). Collectively, these findings indicated that down-regulation of PHB2 would inhibit the proliferation of RD cells, suggesting that PHB2 is required for RD cell proliferation. Interestingly, knockdown of PHB2 expression in normal rat myoblast L6 cells and normal liver L02 cells did not significantly affect the cell proliferation (see Supplementary Figs S3 and S4).

### PHB2 knockdown results in cell cycle arrest and apoptosis in RD cells

**Figure 2:**
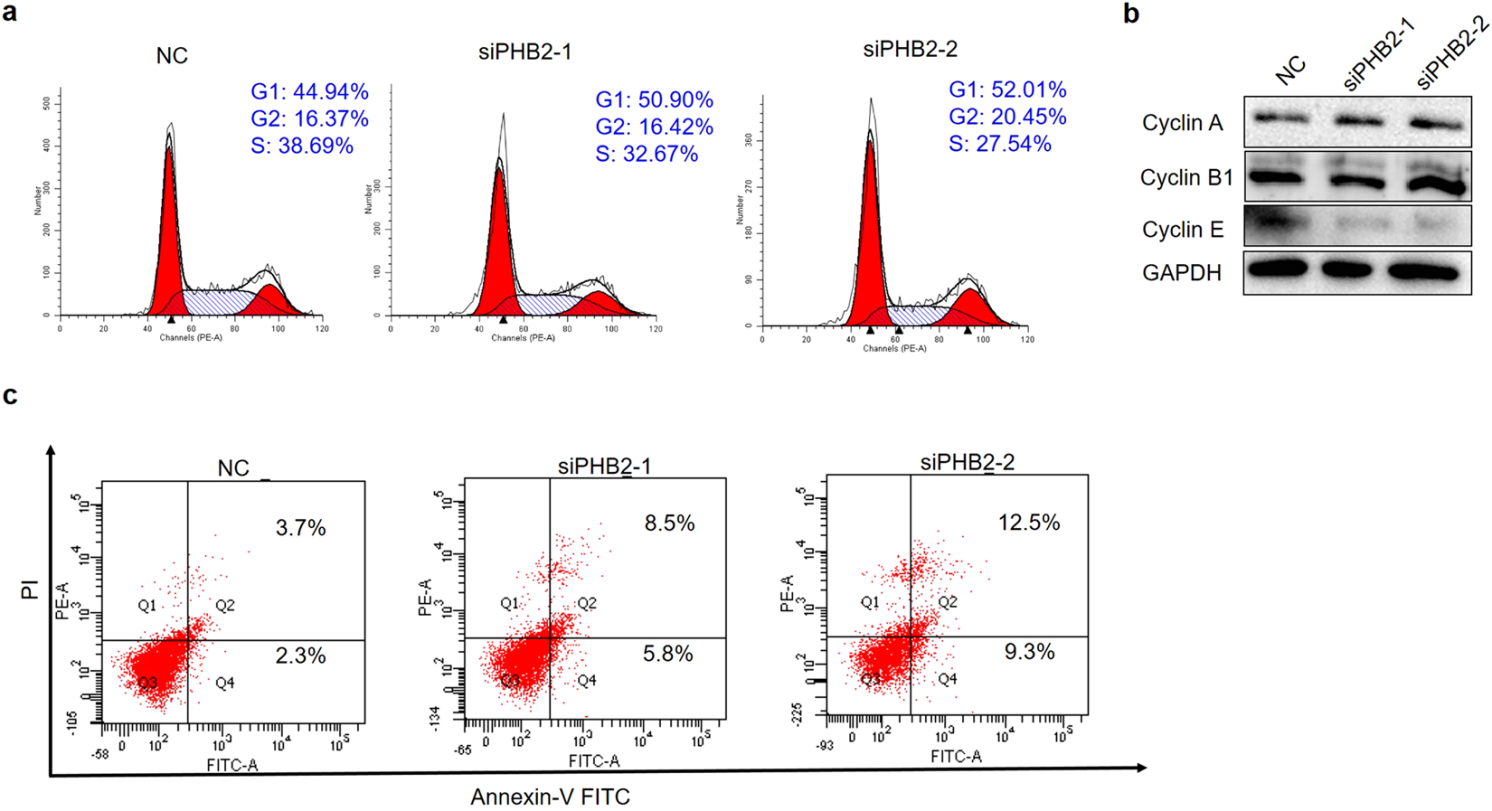
PHB2 knockdown results in cell cycle arrest and apoptosis in RD cells. (**a**) Cell cycle analysis by flow cytometry. Cells were transfected with siRNAs. After 48 h, RD cells were fixed and stained with propidium iodide (PI), and DNA content was detected using flow cytometry. Results are representative of three independent experiments. (**b**) Detection of levels of cell cycle-related proteins. RD cells were transfected with siRNAs. Forty-eight hours later, cells were lysed and subjected to Western blotting, and GAPDH was detected as a loading control. Full-length blots are presented in Supplementary Fig. S1. Results are representative of three independent experiments. (**c**) Flow cytometric analysis of apoptosis. RD cells were transfected with siRNAs. Forty-eight hours later, cells were stained with both PI and annexin V, followed by flow cytometric analysis. Results are representative of three independent experiments.

The cell cycle plays a key role in cell proliferation. Thus, we further analyzed the cell cycle of siRNA-treated RD cells by using flow cytometry in order to evaluate whether the inhibition of RD cell proliferation by PHB2 siRNAs was achieved by arresting the cell cycle. As shown in Fig. 2a, a higher proportion of cells transfected with PHB2 siRNAs was arrested in the G1 phase compared with negative control cells. Upon transfection with siPHB2-1 and siPHB2-2, the percentage of G1 phase cells increased to 50.90% and 52.01%, respectively (Fig. 2a). To investigate the underlying molecular mechanisms, we examined various cyclins which are key cell cycle regulators. The results showed that PHB2 knockdown reduced the expression of cyclin E (Fig. 2b), while it had no obvious effect on the levels of cyclin A and cyclin B1. Cyclin E plays an essential role in the G1-S transition. Therefore, these results validated that PHB2 silencing could indeed induce cell cycle arrest at the G1 phase. Next, we tested the possibility of inducing apoptosis by PHB2 siRNAs. We found that although apoptosis was not induced in a large number of RD cells, transfection with PHB2 siRNAs resulted in a slight but stable increase in the proportion of those cells undergoing apoptosis compared with negative control cells (3.7% vs 8.5% or 12.5%) (Fig. 2c). Thus, the decreased expression of PHB2 inhibited the proliferation of RD cells possibly by impairing cell cycle and inducing cell apoptosis.

### PHB2 knockdown promotes RD cell differentiation

**Figure 3:**
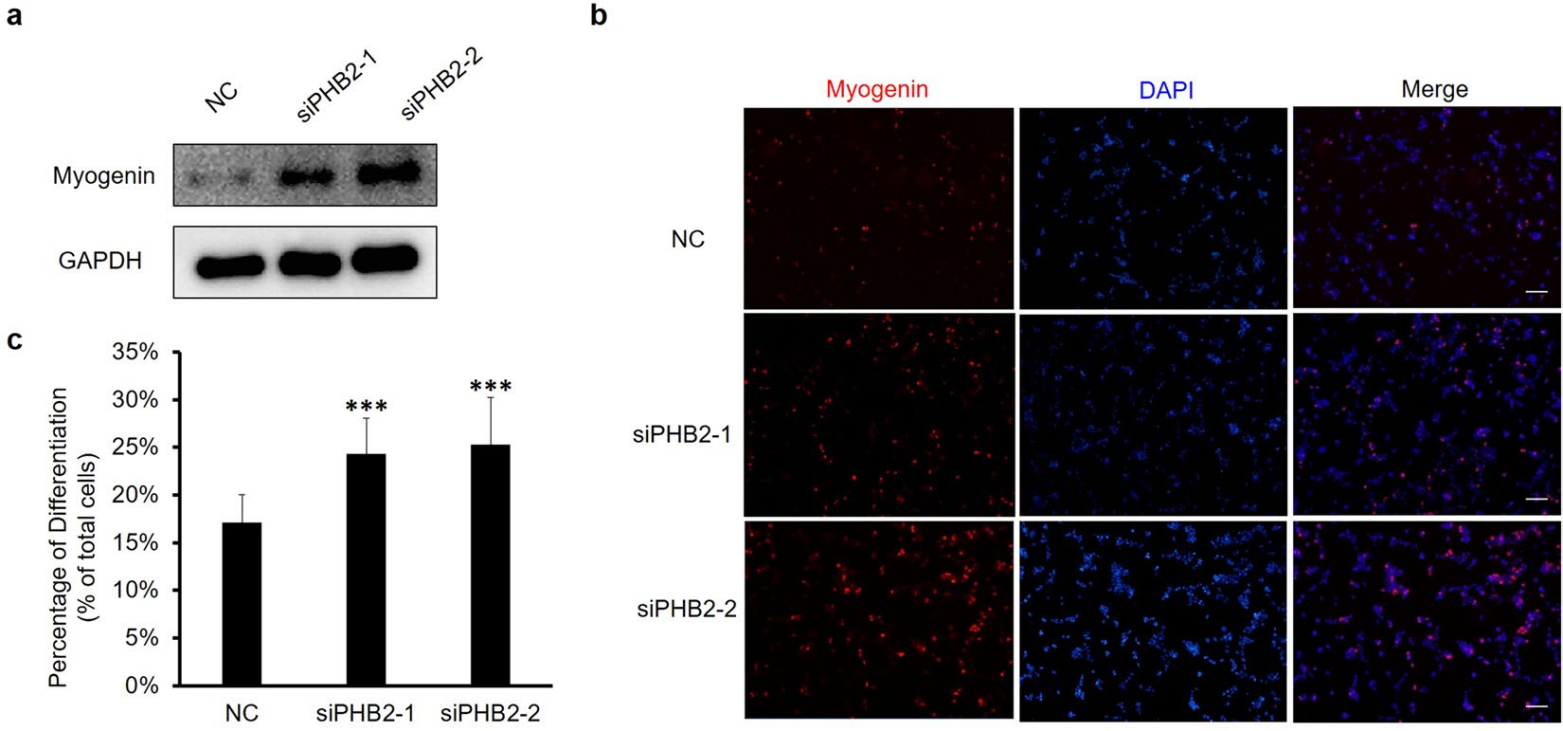
PHB2 knockdown promotes RD cell differentiation. (**a**) Detection of myogenin by Western blot. RD cells were transfected with siRNA and the growth medium was replaced 6 hours later. The next day, the medium was replaced with the differentiation medium (2% calf serum, H-DMEM). Cell extracts were subjected to immunoblotting with indicated antibodies, and GAPDH was detected as a loading control. Full-length blots are presented in Supplementary Fig. S1. (**b**) Immunofluorescence staining of myogenin in RD cells. Forty-eight hours after transfection, RD cells were fixed and subjected to immunostaining for myogenin (red). The nuclei of cells were counterstained with DAPI (blue). *Scale bar* 200 μm. Results are representative of three independent experiments. (**c**) Quantitative analysis of myogenin in panel B. Data are presented as the mean ± S.D. (n = 3), *P < 0.05, **P < 0.01, ***P < 0.001, two-tailed Student’s t test.

The clinical outcome of RMS is associated with the degree of muscle differentiation, and greater expression of myogenic transcriptional factors indicates a better prognosis. We wondered whether PHB2, as an inhibitor of myogenic transcription factor and muscle differentiation, is linked to differentiation defects in RMS. Therefore, we investigated the effect of PHB2 knockdown on the differentiation of RD cells. Myogenin is a transcriptional factor critical for differentiation of skeletal muscle and also designated as an early myogenic differentiation marker. The lowered expression of PHB2 was observed to lead to the higher expression of myogenin to some extent (Fig. 3a). We further detected myogenin by immunofluorescence staining in RD cells. Consistent with the immunoblotting results, the percentage of myogenin-positive cells increased in PHB2-silenced RD cells, compared with the negative control (Fig. 3b and c). Altogether, these results indicated that PHB2 might participate in maintaining the low degree of differentiation in RD cells.

### PHB2 is partially localized in the nucleolus in RD cells

**Figure 4:**
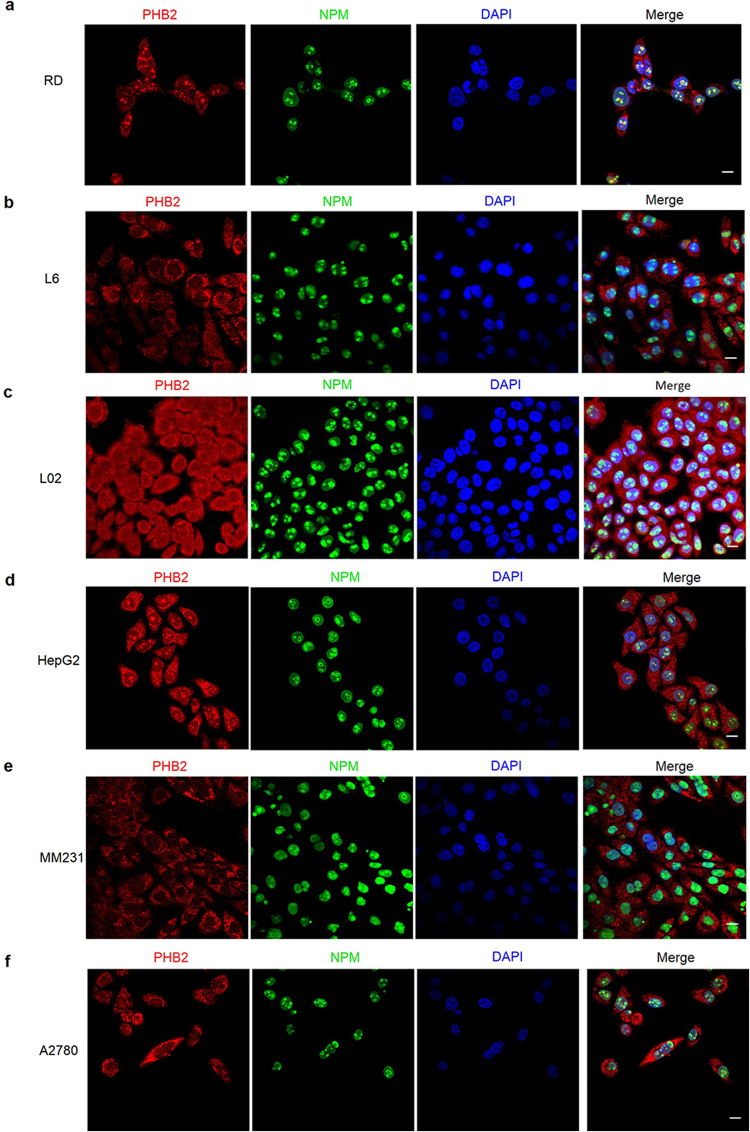
PHB2 is partially localized in the nucleolus in RD cells. (**a–f**) Cell lines including RD (**a**), L6 (**b**), L02 (**c**), HepG2 (**d**), MDA-MB-231 (MM231) (**e**) and A2780 (**f**) cells were fixed for double immunofluorescence staining of PHB2 (red) and NPM (green). The nuclei of the cells were counterstained with DAPI (blue). The co-localization of PHB2 and NPM is illustrated by the merged images. Results are representative of three independent experiments. *Scale bar* 20 μm.

To further delineate the biological function of PHB2 in RD cells, we evaluated the subcellular localization of PHB2 in RD cells. By using immunofluorescence, we observed that aside from the expected mitochondrial localization, a substantial amount of PHB2 seemed to be localized in the nucleolus of RD cells. To further confirm this new observation, we examined the cellular co-localization of PHB2 and nucleophosmin (NPM, also known as B23, a nucleolar protein) by immunofluorescence staining and confocal microscopy. PHB2 showed an obvious co-localization with NPM in RD cells. NPM is a phosphoprotein localized in granular regions of the nucleolus, which is involved in diverse biological functions including genomic stability, tumorigenesis and ribosome biogenesis^16–18^. This result confirmed that PHB2 is located in the nucleolus of RD cells (Fig. 4a). Consistently, partial nucleolar localization of PHB2 was also observed in two other ERMS cell lines, A204 and TE671, and one ARMS cell line RH30 (Supplementary Fig. S5). Interestingly, in normal cells, such as L6 and L02 cells, PHB2 was mainly localized in the cytosol/mitochondria and hardly observed in the nucleolus (Fig. 4b and c). We then extended the co-localization analysis to other human cancer cell lines, such as HepG2, MDA-MB-231 (MM231) and A2780. The nucleolar localization of PHB2 was also observed in HepG2 cells (Fig. 4d), but not in MM231 and A2780 cells (Fig. 4e and f). These data indicated that PHB2 might elicit unique regulatory functions in RD cells via its specific subcellular localization to the nucleolus.

### PHB2 silencing alters morphology of the nucleolus in RD cells

**Figure 5:**
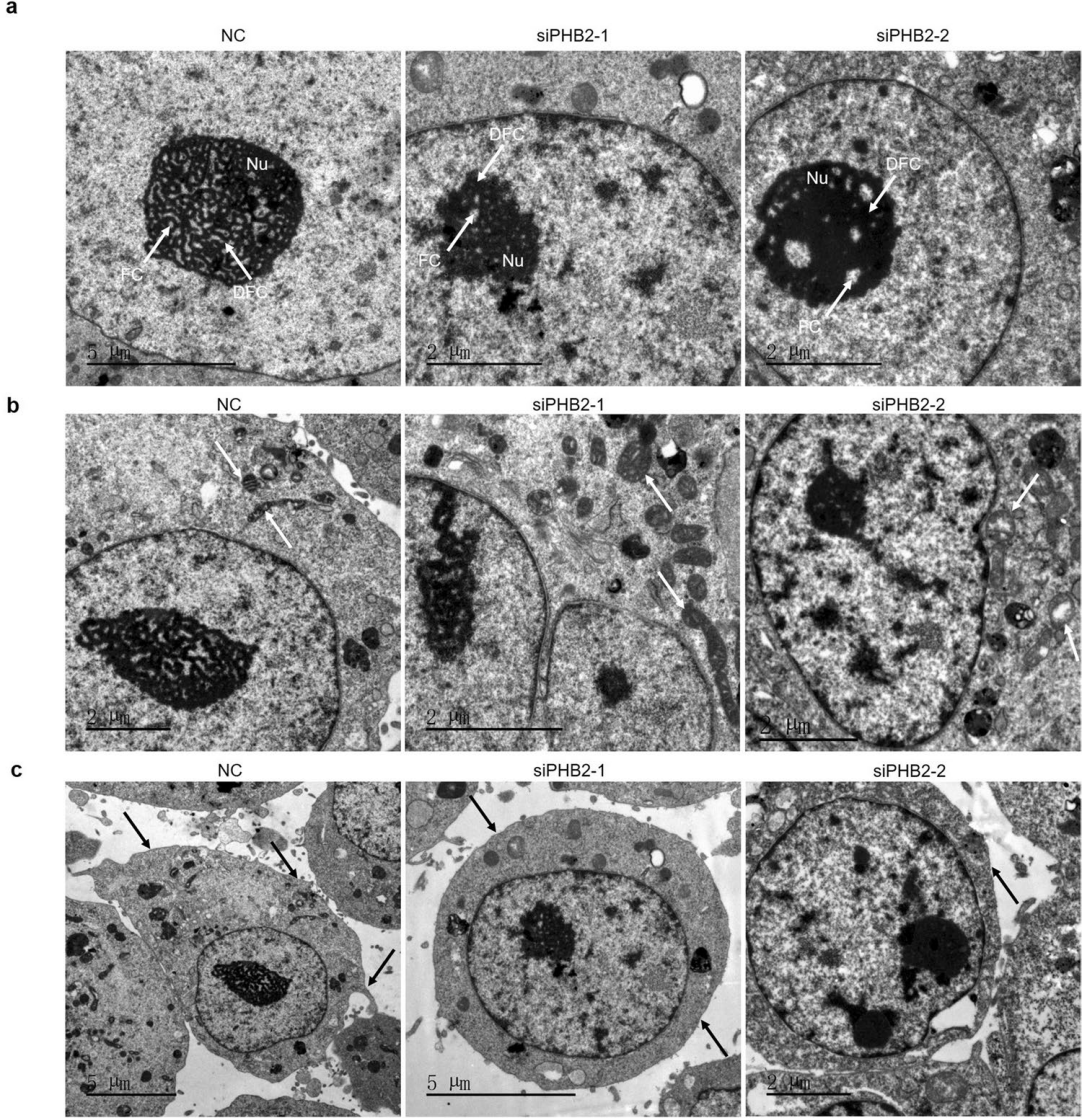
Morphological changes of nucleolus in PHB2 knockdown RD cells by transmission electron microscopy (TEM). (**a**) Structural features of the nucleolus (Nu) in RD cells. Forty-eight hours after RD cells were transfected with siRNAs, they were collected, fixed and then submitted for TEM observation. Arrows indicate areas of FC and dense DFC in the nucleolus. (**b**) Mitochondria alterations in the same batch of RD cells as in A. White arrow heads point to mitochondria in RD cells. (**c**) Cell membrane changes of the same batch of RD cells as in A. Black arrows point to the cell membrane.

Since a considerable amount of PHB2 appeared in the nucleolus of RD cells, we speculated that it may be involved in regulation of nucleolar morphology and function. To test this hypothesis, we observed the subcellular structures of siRNA-transfected RD cells by using transmission electron microscopy (TEM). As expected, a series of alterations could be found in the morphology and architecture of the nucleolus in PHB2 knockdown RD cells compared with negative control cells. The nucleolus in mammalian cells has three primary structures with morphologically and functionally distinct components: fibrillar centers (FCs), dense fibrillar component (DFC) and granular component (GC)^19^. FCs house the bulk of the rDNA. The synthesis of pre-ribosomal RNA (rRNA) executed by RNA polymerase takes place at the interface between FCs and DFCs^20,21^. Early pre-rRNA cleavages occur in DFC territory^22^, and further processing is at the GC^23^. In our study, we observed that PHB2 knockdown led to a series of alterations in the nucleolus, i.e. increased nucleolar density, disappearance of sponge-like nucleolus, irregular nucleoli structure, fuzzy nucleolus boundaries and nucleolus contraction (Fig. 5a). These changes could partly explain the inhibitory effect of PHB2 silencing on RD cell proliferation. Aside from these alterations in the nucleolus of RD cells, PHB2 knockdown also led to the damage of mitochondria, as previously reported, such as loss of structural integrity of mitochondria, and cristae becoming intumescent and intricately stacked (Fig. 5b). In addition, we also found that PHB2 silencing induced a more uniform cell boundary and fewer cell microvilli (Fig. 5c).

Collectively, these data indicate that PHB2 deficiency by siRNA treatment in RD cells impaired the ultrastructure of the nucleolus and mitochondria, likely contributing to the inhibition of RD cell proliferation and subsequent inhibition of the tumorigenicity of these cancer cells.

### PHB2 silencing suppresses rRNA transcription, reduces occupancy of c-Myc at rDNA and facilitates MyoD binding to rDNA

**Figure 6:**
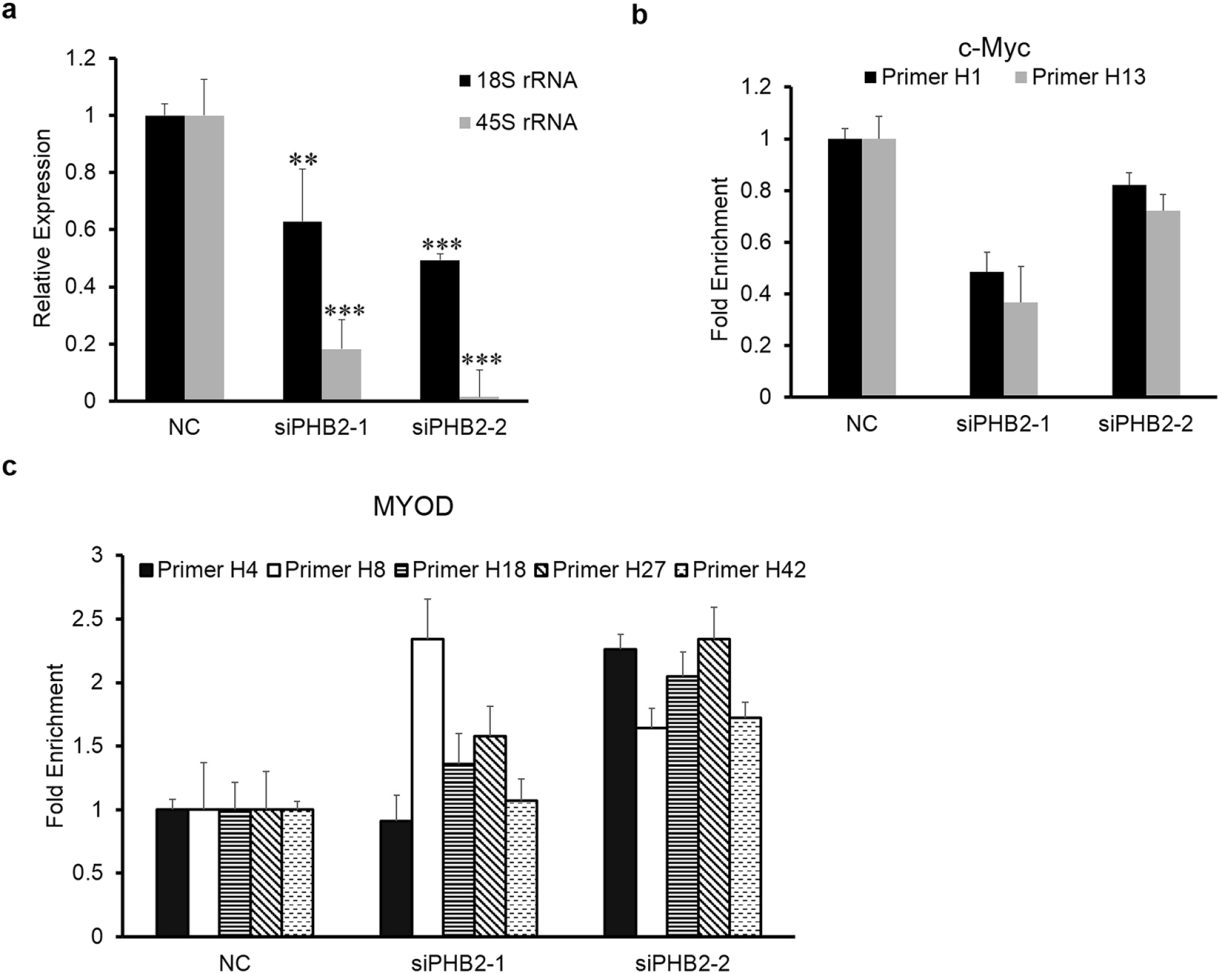
PHB2 knockdown inhibits 45S rRNA and 18S rRNA transcription and reduces occupancy of c-Myc on rDNA, but facilitates MyoD binding to rDNA in RD cells. (**a**) The 45S rRNA and 18S rRNA in RD cells were amplified by conventional SYBR real-time PCR analysis. After transfection, RNA extraction was performed. cDNA was prepared from total RNA using the TransScript First-Stand cDNA Synthesis SuperMix with random primers. Data are presented as the mean ± S.D. (*n* = 3), *P < 0.05, **P < 0.01, ***P < 0.001, two-tailed Student’s *t* test. (**b**) ChIP analysis of Myc binding to rDNA in RD cells transfected with siRNA. RD cells were transfected with PHB2 siRNA, collected and fixed after 48 h. Immunoprecipitated rDNA was quantitated by real-time PCR with two primer sets, H1 and H13. Data are presented as the mean ± S.D. (*n* = 3). (**c**) RD cells were prepared as in panel B, and then the ChIP assay of MyoD binding to rDNA was conducted. qPCR analysis of MyoD was conducted to check the quality of the ChIP-DNA, and five primer sets targeted to the rDNA E-box (H4, H8, H18, H27, H42) were utilized. Data are presented as the mean ± S.D. (*n* = 3).

rRNA is the RNA component of the ribosome, participating in ribosomal biogenesis. Transcription and processing of most rRNAs occur in the nucleolus. To investigate whether PHB2 is involved in regulating nucleolar function, we examined the effect of PHB2 knockdown on transcription of rDNA in the nucleolus. We detected the level of 45S rRNA, a pre-rRNA transcribed by RNA polymerase I, and its mature 18S rRNA product by quantitative real-time RT-PCR (RT-qPCR) in siRNA-treated RD cells. The results showed that both 45S pre-rRNA and 18S rRNA were down-regulated in siPHB2-transfected RD cells compared with control cells (Fig. 6a). These data suggest that rRNA gene transcription was suppressed in siPHB2-transfected RD cells.

The above results indicated that nucleolus-localized PHB2 might participate in regulation of the rRNA transcription in RD cells. To further clarify the possible underlying mechanisms, we investigated the effect of PHB2 knockdown on the binding of c-Myc to the rDNA promoter. The c-Myc oncogene is one of the well-known transcription factors that activates rDNA transcription in response to mitogenic signals during proliferation^24,25^. The rDNA repeats contain numerous canonical and non-canonical E-boxes [element recognized by basic helix-loop-helix (bHLH) transcription factors including c-Myc]^24^. We therefore used several sets of primer pairs^25^ to perform a chromatin cross-linking and immunoprecipitation (ChIP) assay. As shown in Fig. 6b, PHB2 silencing decreased the enrichment of rDNA immunoprecipitated with anti-c-Myc antibodies, which may have contributed to the reduced rRNA transcription. In addition, our previous study showed that PHB2 could interact with the bHLH transcription factor MyoD and repress MyoD-dependent gene transcription in myoblasts^15^. However, aside from catalyzing the RNA polymerase II-mediated muscle-specific gene transcription, Ali *et al*. also demonstrated that MyoD could occupy rDNA loci and suppress rRNA expression during skeletal muscle lineage progression, concomitant with decreased rRNA expression and reciprocal loss of occupancy by c-Myc^26^. Therefore, we further investigated the occupancy of MyoD on rDNA in siRNA-transfected RD cells. Results of the ChIP assay with an anti-MyoD antibody showed that the occupancy of MyoD on rDNA repeats exhibited a significant increase when PHB2 was silenced (Fig. 6c). Taken together, our data indicate a potential PHB2-dependent mechanism which inhibits myogenic differentiation and facilitates proliferation through promoting c-Myc activation of rDNA transcription, while also inhibiting MyoD binding to rDNA at least in RD cells.

## Discussion

RMS is the most common soft-tissue sarcoma in children and adolescents, which is a mesenchymal malignancy arising as a consequence of regulatory disruption of skeletal muscle progenitor cell growth and differentiation. As a result, RMS cells are unable to undergo full-scale terminal myogenic differentiation despite the expression of MyoD and myogenin, two important factors required for myogenic cell differentiation^27–29^. PHB2, a highly evolutionarily conserved protein, has been demonstrated to possess multiple biological activities, including transcriptional regulation, nuclear signaling, mitochondrial structural integrity maintenance and cell division modulation in various cellular compartments^30^. Here, using the RD cell line, we first showed the localization of PHB2, a repressor of skeletal muscle differentiation, in the nucleolus where it participated in regulation of Myc-mediated rRNA synthesis, which may serve as a mechanism to facilitate cell proliferation while inhibiting differentiation.

The involvement of PHB2 in the regulation of cancer cell proliferation has been reported. However, its effects are debated in cancer cells of different origins. In estrogen receptor (ER)-positive breast cancer, PHB2 acts as a tumor inhibitor to suppress the growth of breast cancer cells by repressing the estrogen/ER signaling pathway^31^. Meanwhile, in hepatocellular carcinoma, PHB2 is required for cancer cell proliferation^32^. In the present study, we found that silencing of PHB2 could obviously inhibit RD cell proliferation and survival (Fig. 1). Furthermore, we observed that upon PHB2 silencing, RD cells showed a stable tendency of cell cycle G1 phase arrest. These results are consistent with the study by Equilibrina *et al*., which demonstrated that PHB2 was required for G1/S progression since the number of cells undergoing extended G1 phase increased significantly under the PHB2 knockdown condition^33^. We also found that PHB2 silencing could induce apoptosis in a small proportion of RD cells. This result is in agreement with a previous report showing that PHB2 knockdown in Hela cells resulted in apoptosis^34^. However, we did not find that PHB2 silencing could affect the migration and invasion of RD cells (data not shown), which disagrees with previous findings^13^. Therefore, the precise activities of PHB2 in these cellular processes are worth further study.

Aside from supporting RD cell proliferation, PHB2 might also play a role in the differentiation state of these cells. Our results showed that PHB2 silencing resulted in a higher level of myogenin expression in RD cells (Fig. 3) and an increased proportion of myogenin-positive RD cells. These results are expected since we previously demonstrated that PHB2 could act as a repressor for muscle differentiation by inhibiting the transcriptional activities of MyoD and MEF2^15^. Although RMS cells express multiple essential myogenic transcription factors such as MyoD, MEF2 and myogenin, they cannot fully differentiate^35^. The uncontrolled repressive activity of PHB2 may partly contribute to the defective differentiation state of RMS cells. In other tumors, the role of PHB2 in maintaining the poorly differentiated state has not been reported thus far. Notably, the differentiation enhancement by PHB2 silencing on RD cells was not as significant as expected. We speculate that the main role of PHB2 in RD cells is to regulate cell proliferation, although we cannot exclude the possibility that the results were affected by low siRNA transfection efficiency.

PHB2 is located in different cellular compartments to elicit different functions^36^. In inner mitochondrial membranes, PHB2 (by interacting with PHB1) maintains the structure of mitochondria and the integrity of mitochondrial respiration machinery^37,38^. In the nucleus, PHB2 is involved in transcription regulation by interacting with multiple transcription factors, including estrogen receptor, MEF2, MyoD^15,34,39^. In the plasma membrane, PHB2, as a receptor-associated protein, is required for insulin-like growth factor binding protein (IGFBP)-6 induced cell migration of RMS cells^13^. PHB2 in the mitochondria is found dominantly and ubiquitously in almost in all tested cells, while other subcellular localizations may be dependent on the type or given stage of cells and are usually restricted to only a small fraction of PHB2 molecules. Here, we identified a new pattern of subcellular localization in RD cells by observing quite a few PHB2 molecules in the nucleolus (Fig. 4). Meanwhile, we further found that PHB2 also localized in the nucleolus in some other examined tumor cells, but a clear nucleolar localization of PHB2 was not observed in normal myoblasts L6 cells, normal liver L02 cells and several types of tumor cells (MM231 and A2780). Thus, mechanisms that mediate PHB2 localization in the nucleolus or other cellular compartments are worth deeper investigation.

The nucleolus, as the most prominent nuclear substructure, is the center of rDNA transcription and facilitates ribosome synthesis. rDNA transcription is important for malignant cell growth and proliferation because the cell must increase ribosome biogenesis to meet the high demand of protein synthesis. Increased sizes and numbers of nucleoli have been recognized as hallmark features of most of tumor types^40^. Recently, increased ribosome biogenesis, which is an important event of tumorigenesis and cancer progression^41^ accompanied by an increased rate of rRNA synthesis in the nucleolus, has become a hallmark of cellular proliferation^42^. Therefore, aberrant nucleolar substructure and function are closely related to cancer progression. Our data showed that PHB2 silencing led to smaller and more dense nucleoli compared with the negative control (Fig. 5). Accordingly, upon PHB2 knockdown, the transcription of rDNA was repressed, and the rRNA level clearly declined (Fig. 6). These results suggest that PHB2 directly or indirectly participates in the regulation of the nucleolus function, which may be one of the mechanisms by which PHB2 is involved in regulating cell proliferation.

Myc is an important and direct regulator of rRNA transcription^43^. In this study, we found that PHB2 silencing in RD cells significantly decreased Myc occupancy at the rDNA promoter, which may account for the reduction in rRNA level upon PHB2 knockdown by siRNA. This data indicates that nucleolar PHB2 is required for Myc to regulate the transcription of rRNA at least in RD cells. On the other hand, MyoD, as a phenotypic transcription factor, has been shown to control differentiation of mesenchymal progenitors into myoblasts by suppressing rRNA gene transcription through selectively interacting with rRNA genes^26^. Our previous studies have demonstrated that PHB2 is a binding partner and repressor of MyoD and inhibits the transcriptional activity of MyoD for skeletal muscle specific structural gene expression during myogenesis^15^. Interestingly, here we also found that down-regulation of PHB2 in RD cells enhanced the binding of MyoD to the promoter of rDNA (Fig. 6), suggesting that PHB2 not only represses the transcriptional activity of MyoD but also alleviates the repressive effect of MyoD on rRNA transcription. Therefore, the present data indicate that the repression of MyoD binding to rDNA by nucleolar PHB2 is one of the mechanisms underlying the differentiation defect of RD cells even though MyoD is expressed. Altogether, these results illustrate the functional significance of the nucleolar localization of PHB2 in RD cells. In view of the above results, we hypothesize that PHB2 plays multiple pathophysiological roles in rhabdomyosarcoma. On one hand, it supports proliferation by enhancing the activity of Myc in nucleolus and/or maintaining normal mitochondrial processes. On the other hand, PHB2 inhibits differentiation by repressing the activity of MyoD.

Cellular localization is closely related to their biological functions of proteins. Both mitochondria and nucleolus localized PHB2 can possibly play a role in regulating RD cell proliferation, survival and differentiation. However, our present study did not provide direct evidence to demonstrate the localization–function correlation of PHB2 in RD cells since we could only silence global PHB2 expression and not specifically down-regulate either mitochondrial or nucleolar PHB2. Although a considerable amount of PHB2 does exist in nucleolus of RD cells and the morphology and function of nucleolus were indeed destroyed by PHB2 silencing in RD cells, we could not exclude the possibility that alterations in the PHB2 level of the mitochondria may indirectly influence rDNA expression and nuclear stability. Therefore, future studies are needed to clarify how PHB2 localizes in different cellular compartments and its functional roles in those different compartments. Notably, some of our preliminary data showed diverse regulatory roles of PHB2 in various cell lines. Upon PHB2 silencing, the human embryo liver cell line L02 neither showed a decrease in cell proliferation, nor exhibited cell cycle G1 phase arrest and reduced rDNA expression (Supplementary Fig. S3). Similarly, the proliferation of another normal cell line, rat myoblast L6 cells, was also not affected by PHB2 silencing (Supplementary Fig. S4). Both of these normal cell lines did not show obvious nucleolar PHB2 staining in our study. Meanwhile, cell proliferation in human hepatocarcinoma HepG2 cells and RH30 cells, which are positive for nucleolar PHB2 staining, was significantly inhibited by PHB2 down-regulation to different extents (Supplementary Figs S2a and S5d). However, compared to RD cells, HepG2 cells transfected with PHB2 siRNAs demonstrated no obvious cell cycle arrest, and rDNA expression was only decreased slightly (Supplementary Fig. S2b and c). These preliminary data point to the complicated working mechanisms of PHB2 in cellular processes, which may differ between normal and malignant cells or vary among cells with different tissue origins or in distinct stages. All of these phenomena are worth further investigation.

In conclusion, for the first time our study identified a unique localization for PHB2 in RD and other tumor cells, which further deepens our understanding of this conserved protein and highlights the significance of its function. PHB2 may play a different biological role in different cells or different physiological and pathological conditions. Novel and complex roles of PHB2 are continually brought to light. Wei *et al*. recently found that PHB2 is a crucial mitophagy receptor involved in targeting mitochondria for autophagic degradation^44^. In our study, ectopic nucleolar-localized PHB2 in RMS, as a regulator of rDNA transcription, was found to support Myc-dependent rDNA transcription and maintain nucleolar structure which participates in sustaining the survival and proliferation of RD cells. In addition, PHB2 via the regulation of the access of MyoD to rDNA adopts an additional mechanism to repress MyoD activities and thus keep RD cells less differentiated. Altogether, the findings of our study provide new insight into the pathogenesis of RMS.

## Materials and Methods

### Cell culture

L6, MDA-MB-231 (human breast cancer cells), HepG2, human RD, L02 and A2780 cell lines were purchased from the Chinese Academy of Sciences Shanghai Institute for Biological Sciences-Cell Resource Center. Human RD cells were maintained in GM, composed of high-glucose Dulbecco’s Modified Eagle’s Medium (DMEM) (Gibco, Grand Island, NY, USA) supplemented with 10% fetal bovine serum (FBS, Ausbian, Australia). L6 myoblasts were maintained in α-MEM (Hyclone, Logan, UT, USA) containing 10% FBS as described previously^45^. HepG2 and MM231 cells were cultured in DMEM (Sigma, St. Louis, MO, USA) supplemented with 10% FBS (Sijiqing, Hangzhou, China). A2780 cells were grown in RPMI-1640 (Sigma) supplemented with 10% FBS (Sijiqing). L02 cells were grown in RPMI-1640 (Sigma) supplemented with 20% FBS (Sijiqing). All of the cells were maintained under standard conditions (37 °C and 5% CO_2_ in incubator) in the presence of 200 U/ml gentamycin sulfate (Huangzhong Pharmaceutical Co., Ltd., Xiangyang, China).

### Antibodies and reagents

Anti-cyclin A (Cat # sc-751), anti-cyclin B1 (Cat # sc-752), anti-cyclin E (Cat # sc-198), anti-Ki67 (Cat # sc-101861), anti-myogenin (Cat # sc-12732) and anti-MyoD (Cat # sc-304) were from Santa Cruz Biotechnology (Santa Cruz, CA, USA). Anti-PHB2 (Cat # AB10198) was obtained from Millipore (Darmstadt, Germany). Anti-c-Myc (Cat # 13987) were from Cell Signaling Technology (Danvers, MA, USA). Anti-B23 (nucleophosmin/NPM, Cat # B0556) was purchased from Sigma. The mouse monoclonal antibody against GAPDH was purchased from Kangcheng Bio-tech (Shanghai, China). 4,6-diamidino-2-phenylindole (DAPI) and Cell Cycle Analysis Kits were obtained from Beyotime (Shanghai, China). The Cell Proliferation ELISA Kit (5-bromo-20-deoxyuridine, BrdU) was purchased from Roche Diagnostics (Indianapolis, IN, USA). 3-(4,5-dimethylthiazol-2-yl)-2,5-diphenyl-2Htetrazolium bromide (MTT) was purchased from Sigma.

### siRNA transfection

siRNA (100 nM) was transfected into 50–70% confluent RD cells using Lipofectamine 2000 (Invitrogen, Carlsbad, CA, USA) according to the manufacturer’s instructions. The following siRNAs were synthesized at Invitrogen (Shanghai, China): NC (5′-UUCUCCGAACGUGUCACGU-3′), siPHB2-1 (5′-GAAUCGUAUCUAUCUCACA-3′) and siPHB2-2 (5′-CUGAACCCCUCUUGGAUUAAG-3′).

### RNA extraction and RT-qPCR

Total RNA was prepared from cultured cells using Trizol reagent (Invitrogen) following the manufacturer’s instructions. Total RNA was reverse transcribed into cDNA using a real-time PCR kit (TransGen Biotech, Beijing, China). RT-qPCR was performed using 2 × FastStart Universal SYBR Green Master (Rox) (Roche Diagnostics, Mannheim, Germany) according to the manufacturer’s protocol. Cycling conditions were 95 °C for 10 min, followed by 40 cycles of 95 °C for 10 s and 60 °C for 1 min. Sequences for primers used for RT-qPCR are as follows: 18S rRNA (sense: 5′-GCAATTATTCCCCATGAACG-3′, antisense: 5′-GGGACTTAATCAACGCAAGC-3′) and 45S rRNA (sense: 5′-TGTCAGGCGTTCTCGTCTC-3′, antisense: 5′-GAGAGCACGACGTCACCAC-3′). Relative quantification of the expression of each gene was calculated using the △△CT method. All real-time PCR reactions were performed in triplicate.

### Western blotting assay

Western blots were performed as described previously^46^. Briefly, after transfection with siRNAs, total proteins of RD cells were extracted and electro-blotted onto a polyvinylidene fluoride (PVDF) membrane following separation by 12% sodium dodecyl sulfate polyacrylamide gel electrophoresis (SDS-PAGE). The PVDF membrane was sequentially probed with various primary antibodies and horseradish peroxidase (HRP)-conjugated secondary antibodies. Blots were visualized by ECL (Beyotime, Shanghai, China) and imaged by using the MicroChemi bio-imaging system (DNR, Jerusalem, Israel).

### BrdU incorporation assay

RD cells were incubated with 10 µl/well of 10 µM BrdU labeling solution for 6 h at 37 °C. The cells were then fixed with 200 µl FixDenat for 20 min and incubated with 100 µl/well anti-BrdU-POD working solution for 90 min. Subsequently, substrate solution (100 µl/well) was added and incubated for 5–30 min. BrdU uptake was measured at 450 nm using a microplate reader (Bio-Rad, Hercules, CA, USA).

### Flow cytometry analysis

Cell cycle was measured by flow cytometry (FACS Canto, Becton Dickinson, New Jersey, USA) using the Cell Cycle Kit (Beyotime, Shanghai, China) following the manufacturer’s instructions. The cells were fixed with 70% ethanol for 12 h at 4 °C and then incubated with the cell cycle reagent. The results are presented as percentages of cells in different cell cycle phases.

The ratios of apoptotic cells were analyzed using the annexin V-FITC Apoptosis Detection Kit (BD Biosciences, San Jose, CA, USA). The cells transfected with siRNA were harvested, washed twice with PBS, stained according to the manufacturer’s instructions and then analyzed by flow cytometry.

### Immunofluorescence staining

Cells were first fixed in 4% paraformaldehyde for 10 min and then permeabilized with 0.2% TritonX-100 in PBS for 10 min before being blocked with PBS containing 5% bovine serum albumin (BSA) for 30 min. Thereafter, the cells were sequentially incubated with anti-PHB2 and anti-NPM primary antibodies and then with anti-rabbit IgG (H + L) (Alexa Fluor^®^ 647 Conjugate, Cat # 4414, Cell Signaling Technology) and anti-mouse IgG (H + L) (Alexa Fluor^®^ 488 Conjugate, Cat # 4408, Cell Signaling Technology), respectively. The images were acquired using an Olympus FV-1000 confocal laser scanning microscope (Olympus, Tokyo, Japan).

### MTT assay

RD cells transfected with siRNA were seeded in 96-well plates at an initial cell density of 5000 cells per well. After 24 h (defined as the first day), 20 µl of MTT solution (5 mg/ml) was added to each well and incubated for an additional 4 h. The absorbance was measured at 570 nm using a microplate spectrophotometer (Thermo Electron Corporation, Shanghai, China). The experiment was performed on three replicates and repeated three times.

### Clonogenic assay

RD cells transfected with siRNA were plated in 6-well plates at 1500 cells per well in 2 ml of media. After 3 days, cells were washed with fresh media and cultured in 2 ml of fresh media. Ten days later, colonies were fixed in 80% ethanol, stained with 0.1% crystal violet and counted.

### Electron microscopy analysis

RD cells were transfected with indicated siRNAs. Forty-eight hours later, cells were harvested and washed with PBS. The cells were then processed as previously described^47^. Ultrathin sections were examined with the FEI TECNAI SPIRIT electron microscope (Brno, The Czech Republic).

### ChIP and real-time PCR (qPCR) analysis

ChIP assays were performed according to the manufacturer’s instructions [SimpleChIP Plus Enzymatic Chromatin IP Kit (#9005), Cell Signaling Technology]. In brief, to crosslink proteins to DNA, 540 µl of 37% formaldehyde was added to ~4 × 10^6^ cells for 10 min. Cells were lysed and sonicated 5 times for 20 s each time to achieve a chromatin size of 100–150 bp. c-Myc and MyoD were immunoprecipitated using c-Myc and MyoD antibodies, respectively. The ChIPed DNA samples were analyzed with PCR Master Mix for SYBR Green assays (TaKaRa, Beijing, China). Primer pairs used for amplification of E-Box consensus sites on the myogenin gene were as follows. c-Myc primer sets included primer H1 (forward, 5′-GGCGGTTTGAGTGAGACGAGA-3′ and reverse, 5′-ACGTGCGCTCACCGAGAGCAG-3′); primer H13 (forward, 5′-ACCTGGCGCTAAACCATTCGT-3′, and reverse 5′-GGACAAACCCTTGTGTCGAGG-3′). MyoD primer sets included primer H4 (forward, 5′CGACGACCCATTCGAACGTCT-3′ and reverse, 5′-CTCTCCGGAATCGAACCCTGA-3′); primer H8 (forward, 5′-AGTCGGGTTGCTTGGGAATGC-3′ and reverse, 5′-CCCTTACGGTACTTGTTGACT-3′); primer H18 (forward, 5′-GTTGACGTACAGGGTGGACTG-3′ and reverse, 5′-GGAAGTTGTCTTCACGCCTGA-3′); primer H27 (forward, 5′-CCTTCCACGAGAGTGAGAAGCG-3′ and reverse, 5′-CTCGACCTCCCGAAATCGTACA-3′) and primer H42 (forward, 5′-AGAGGGGCTGCGTTTTCGGCC-3′ and reverse, 5′-CGAGACAGATCCGGCTGGCAG-3′). The percentage of ChIPed DNA relative to input was calculated and shown as the mean ± standard deviation (S.D.) from three independent experiments.

### Statistical analysis

Experiments were repeated at least three times. The data was analyzed in Excel; a *t* test was carried out between control and each sample after transfected with siRNA. The significance was set at *P < 0.05, **P < 0.01 and ***P < 0.001. Error bars denote the S.D.

## Electronic supplementary material

Supplementary information
