# Supplementary material for: Prohibitin 2 localizes in nucleolus to regulate ribosomal RNA transcription and facilitate cell proliferation in RD cells
Source: Sci Rep. 2018 Jan 24;8:1479. doi: 10.1038/s41598-018-19917-7 (PMC5784149; doi:10.1038/s41598-018-19917-7)
Supplement: Supplementary file 1 — Supplementary information [file 41598_2018_19917_MOESM1_ESM.pdf]

Prohibitin 2 localizes in nucleolus to regulate ribosomal RNA transcription and facilitate cell proliferation in RD cells

Zilong Zhou[1], Huihan Ai[1], Kun Li[1], Xinlei Yao[1], Wenbin Zhu[2#], Lei Liu[2], Chunlei Yu[2], Zhenbo Song[1], Yongli Bao[1], Yanxin Huang[1], Yin Wu[2], Lihua Zheng[2], Ying Sun[1], Guannan Wang[2], Kewei Ma[3], Luguo Sun[1*] & Yuxin Li[2*]

[1] *National Engineering Laboratory for Druggable Gene and Protein Screening, Northeast Normal University, Changchun 130024, China;* [2] *Institute of Genetics and Cytology, Northeast Normal University, Changchun 130024, China;* [3] *Oncology Center, First Hospital, Jilin University, Changchun 130021;* [#] *Present address: Research Institute of Medical Science and Pharmacy, Qiqihar Medical University, Qiqihar 161006, China;*

*To whom correspondence should be addressed. Email: sunlg388@nenu.edu.cn, liyx486@nenu.edu.cn. Tel: 86-0431-8916-5922; Fax: 86-0431-8916-5917.

Supplementary Figure S1

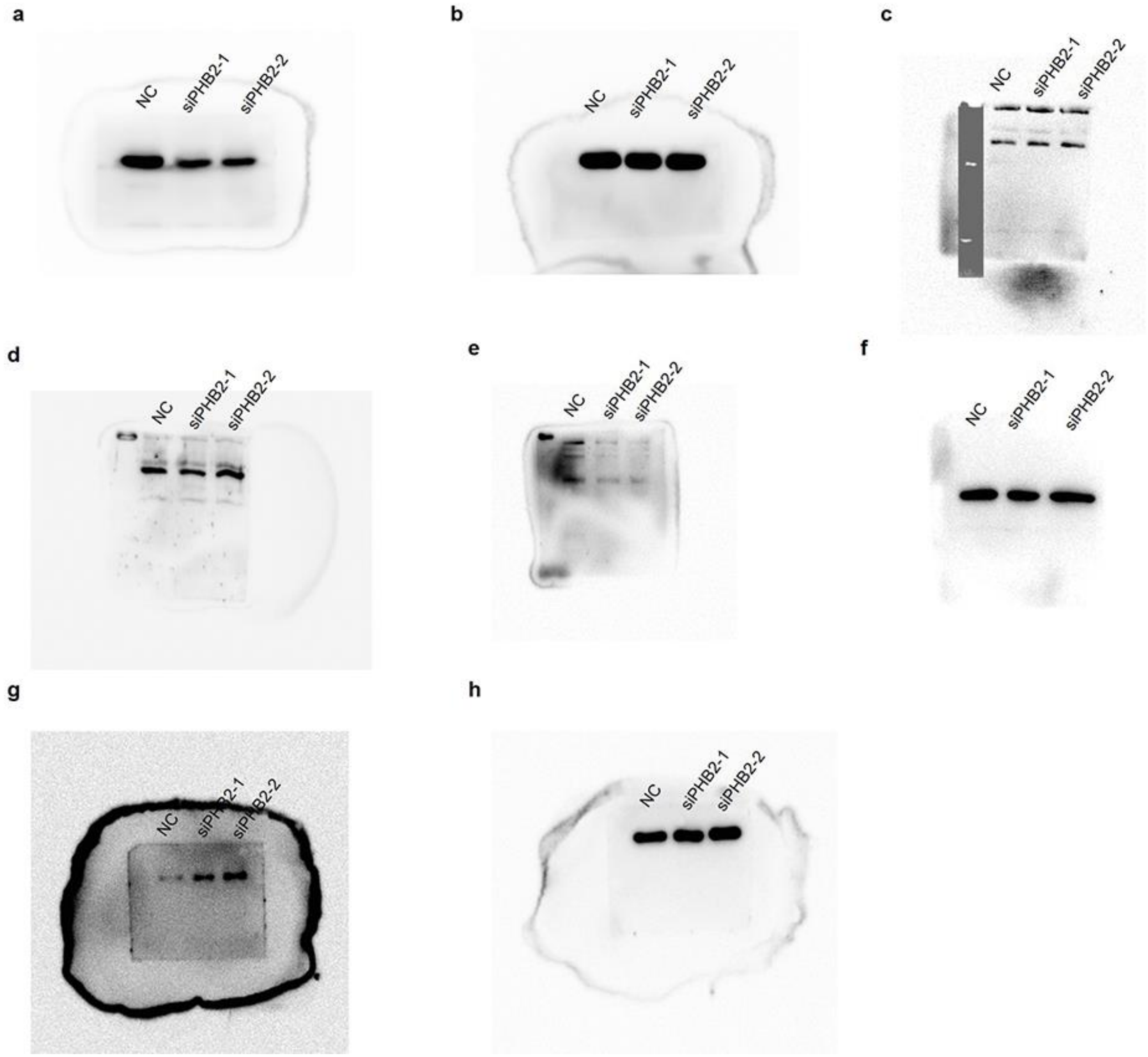

Supplementary Figure S1. Original Western blot images. (a, b) For Figure 1a. a, anti-PHB2 ; b, anti-GAPDH. **(c-f)** For Figure 2b. c, anti-cyclin A; d, anti-cyclin B1; e, anti-cyclin E; f, anti-GAPDH. **(g, h)** For Figure 3a. g, anti-myogenin; h, anti-GAPDH.

Supplementary Figure S2

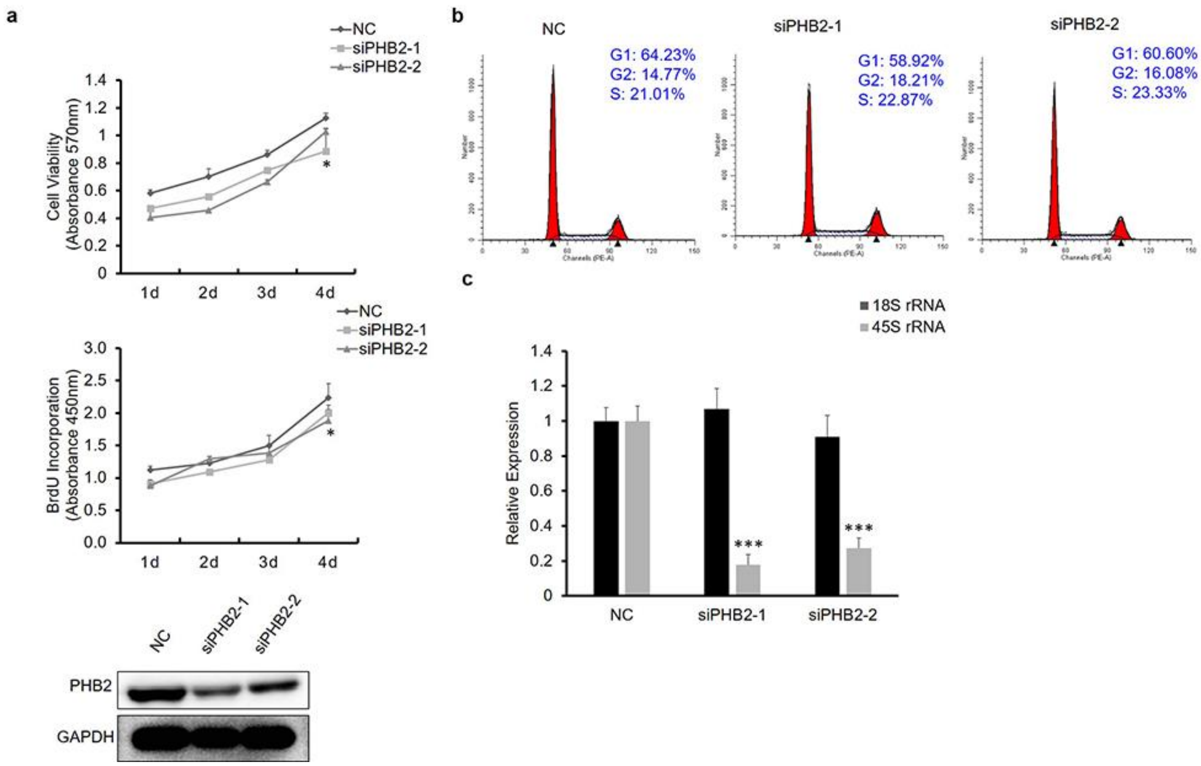

Supplementary Figure S2. Effects of PHB2 silencing in HepG2 cells. (a) Detection of cell proliferation of HepG2 cells with PHB2 silencing. HepG2 cells in a 6-well plate were transfected with siRNAs against PHB2 (siPHB2-1 and siPHB2-2) or negative control (NC) and were then re-plated in triplicate into 96-well plates at 5000 cells per well. Twenty-four hours later, cell viability and proliferation rate were determined by the MTT assay (top), and DNA synthesis was detected by BrdU incorporation (middle). Knockdown efficiency of siRNAs is shown at the bottom of the figure. **(b)** Cell cycle analysis. HepG2 cells were fixed and stained with propidium iodide (PI) 48 hours after siRNA transfection and then subjected to flow cytometric analysis. **(c)** RT-qPCR analysis of rRNA. After siRNA transfection, RNA was extracted, and 45S rRNA and 18S rRNA were quantified by conventional SYBR real-time PCR analysis. Data are presented as the mean ± S.D. (n = 3), *$P < 0.05$, **$P < 0.01$, ***$P < 0.001$, two-tailed Student's t test.

Supplementary Figure S3

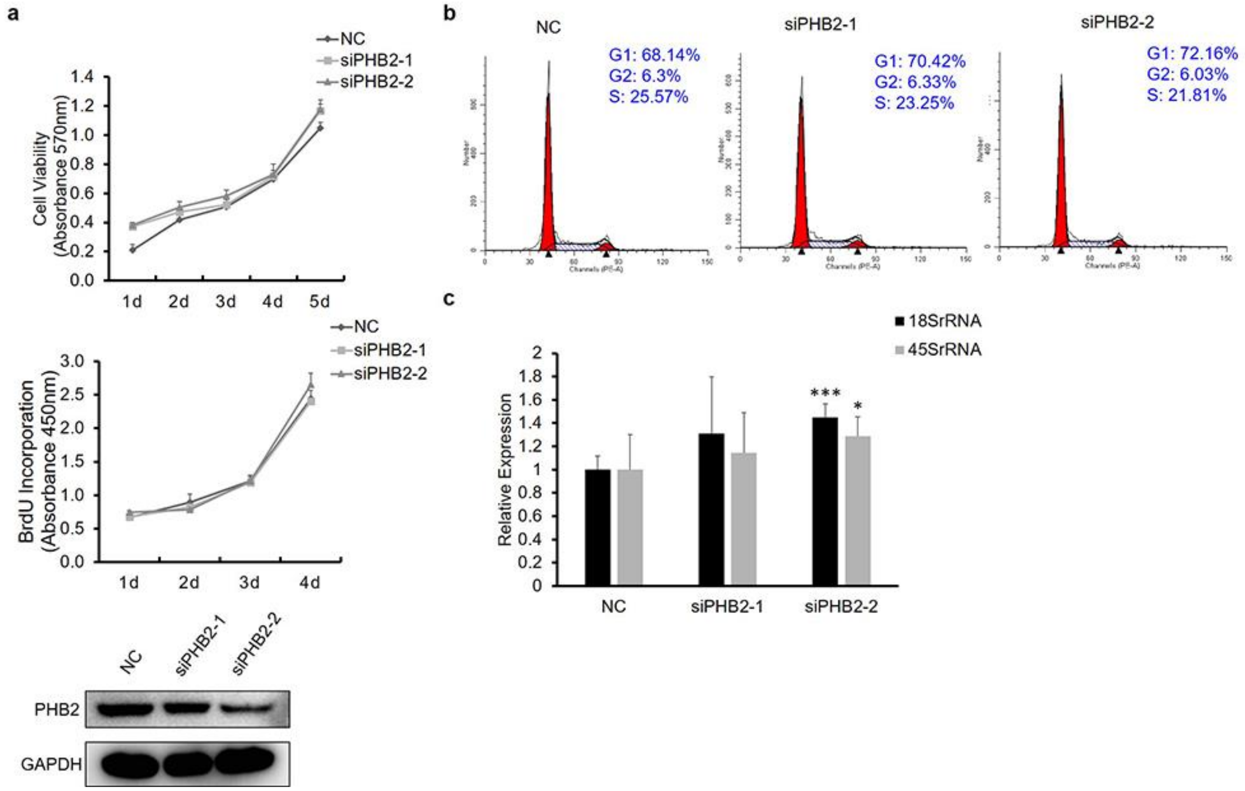

Supplementary Figure S3. Effects of PHB2 silencing in L02 cells. Cells were treated and subjected to analysis as in Figure S2**. (a)** Detection of cell proliferation of L02 cells with PHB2 silencing. Top, MTT assay; middle, BrdU incorporation assay; bottom, knockdown efficiency of siRNAs. **(b)** Cell cycle analysis. **(c)** RT-qPCR analysis of rRNA. Data are presented as the mean ± S.D. (n = 3), *P < 0.05, **P < 0.01, ***P < 0.001, two-tailed Student's t test.

Supplementary Figure S4

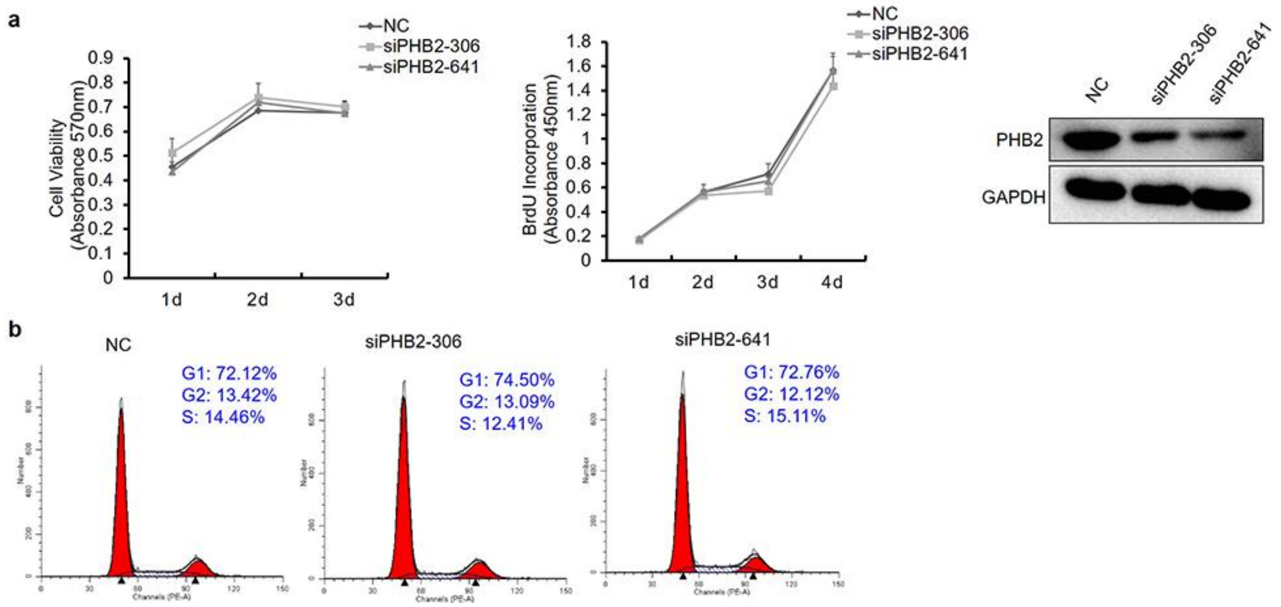

Supplementary Figure S4. Effects of PHB2 silencing in L6 cells. Cells were treated and subjected to analysis as in Figure S2. The siRNAs against rat PHB2 were synthesized at GenePharma (Shanghai, China): siPHB2-306 (5′-GGACACAAUCCUAGCCGAA-3′), siPHB2-641 (5′-CCAAGGACUUCAGCCUCAU-3′) and control siRNA (NC) (5′-UUCUCCGAACGUGUCACGU-3′). **(a)** Detection of cell proliferation of L6 cells with PHB2 silencing. Left, MTT assay; middle, BrdU incorporation assay; right, knockdown efficiency of siRNAs. **(b)** Cell cycle analysis. Data are presented as the mean ± S.D. (n = 3), **P* < 0.05, ***P* < 0.01, ****P* < 0.001, two-tailed Student’s t test.

Supplementary Figure S5

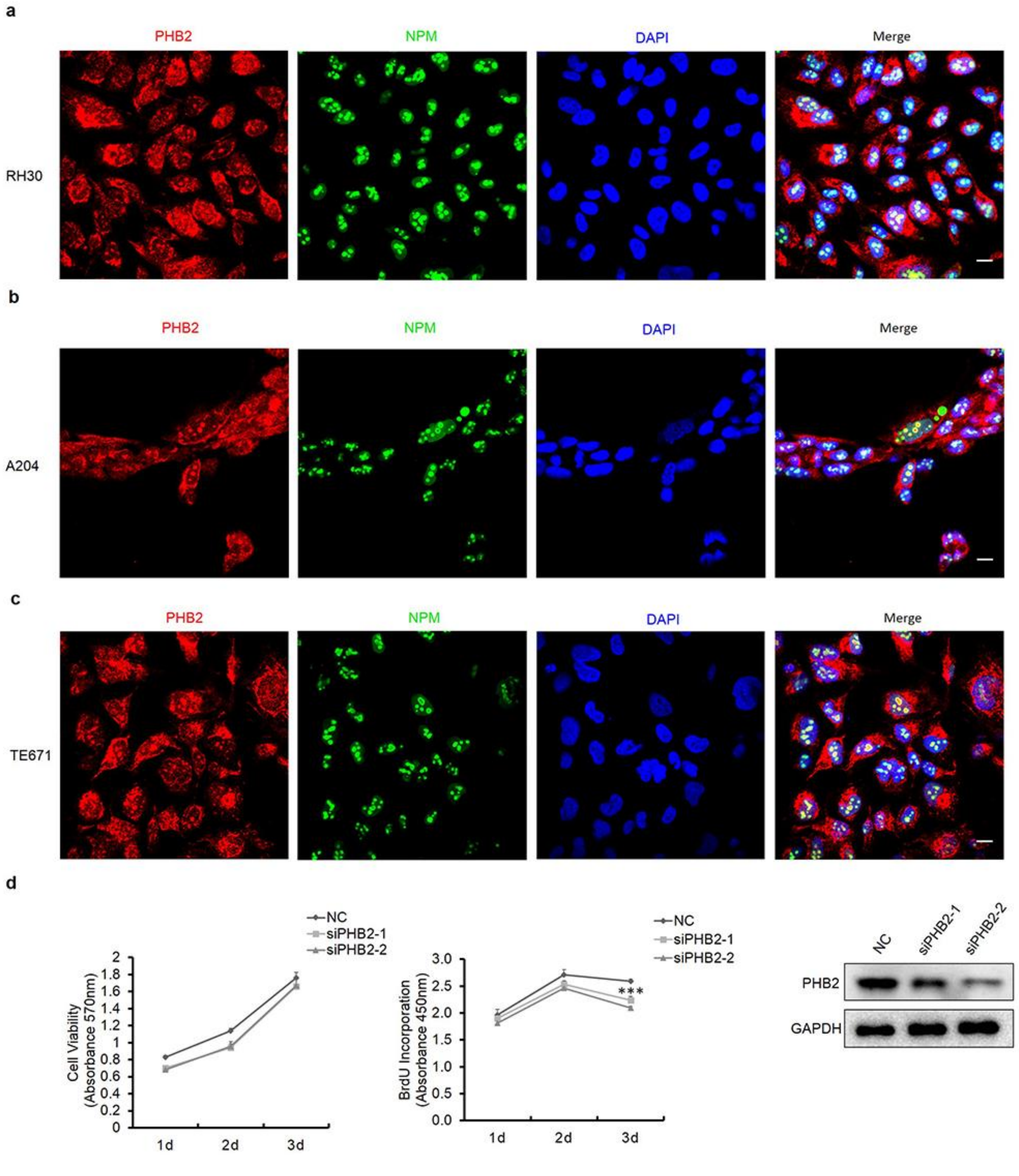

Supplementary Figure S5. The localization of PHB2 in other RMS cell lines. (a-c) Immunofluorescence staining of PHB2 and NPM. RH30 (a), A204 (b) or TE671 (c) cells were fixed for double immunofluorescence staining of PHB2 (red) and NPM (green). Nuclei of the cells were counterstained with DAPI (blue). The co-localization of

PHB2 and NPM is illustrated by the merged images. Results are representative of three independent experiments. *Scale bar* 20 μm. RH30, A204 and TE671 cells were purchased from BeNa Culture Collection (Beijing, China). RH30 and TE671 cells were maintained in high-glucose Dulbecco's Modified Eagle's Medium (DMEM) (Gibco, Grand Island, NY, USA) supplemented with 10% fetal bovine serum (FBS, Ausbian, Australia). A204 cells were cultured in McCoy's 5a (Gibco, Grand Island, NY, USA) supplemented with 10% FBS. **(d)** Detection of cell proliferation of RH30 cells with PHB2 silencing. Cells were treated and subjected to analysis as in Supplementary Figure S2a. Left, MTT assay; middle, BrdU incorporation assay; right, knockdown efficiency of siRNAs. Significance was set at $*P < 0.05$, $**P < 0.01$, $***P < 0.001$, two-tailed Student's t test. Data are presented as the mean ± S.D. (n = 3). Results are representative of three independent experiments.
